# Patient violence towards their family carers: A qualitative exploration of carers’ experiences in psychosis

**DOI:** 10.1192/j.eurpsy.2022.318

**Published:** 2022-09-01

**Authors:** E. Wildman, E. Kuipers, D. Macmanus, J. Onwumere

**Affiliations:** 1 King’s College London, Institute of Psychiatry, Psychology, and Neuroscience, Department Of Psychology, London, United Kingdom; 2 Bethlem Royal Hospital, South London And Maudsley Nhs Foundation Trust, London, United Kingdom; 3 King’s College London, Department Of Forensic And Neurodevelopmental Science, London, United Kingdom

**Keywords:** Carers, violence, aggression, Psychosis

## Abstract

**Introduction:**

Compared to the general population, people living with schizophrenia spectrum disorders (SSD) are more likely to perpetrate acts of violence. When this happens, family members (informal carers) are most commonly the victims. However, family violence by people with SSD is often a taboo topic and largely neglected within public discourse, research, and clinical domains. Consequently, our understanding of families’ experiences and support needs are limited.

**Objectives:**

To develop a detailed understanding of the subjective experiences, and impact, of patient violence towards family carers.

**Methods:**

Individual semi-structured interviews were held with family carers of adults with SSD and a history of violence perpetration towards their family carer. Interview data were subject to thematic analyses using NVivo software.

**Results:**

Twenty-one UK based carers that were predominately White British (90%) and female (81%) were interviewed. Key themes highlight the range of physical and mental injuries endured by carers following patient violence, and speak to carers’ experiences of suffering, living in a constant state of hypervigilance, as well as social isolation in the context of shame, stigma, and an absence of professional and informal support.

**Conclusions:**

Family violence by people living with SSD can and does happen. Yet, too often, carers are left with no option but to continue supporting their relative in the absence of support, even in contexts where this compromises their own safety. The devastating impact of violence is far-reaching, across all levels of the family-system. The findings highlight the danger of neglecting family violence by people with SSD in research and clinical fields.

**Disclosure:**

No significant relationships.

